# Protection of photosystem I during sudden light stress depends on ferredoxin:NADP(H) reductase abundance and interactions

**DOI:** 10.1093/plphys/kiab550

**Published:** 2021-11-22

**Authors:** Melvin Rodriguez-Heredia, Francesco Saccon, Sam Wilson, Giovanni Finazzi, Alexander V Ruban, Guy T Hanke

**Affiliations:** 1 Department of Biochemistry, Queen Mary University of London, London E1 4NS, UK; 2 Laboratoire de Physiologie Cellulaire et Végétale, UMR 5168, Centre National de la Recherche Scientifique (CNRS), Commissariat à l’Energie Atomique et aux Energies Alternatives (CEA), Université Grenoble Alpes, Institut National de Recherche Agronomique (INRA), Institut de Recherche en Sciences et Technologies pour le Vivant (iRTSV), CEA Grenoble, F-38054 Grenoble cedex 9, France

## Abstract

Plant tolerance to high light and oxidative stress is increased by overexpression of the photosynthetic enzyme Ferredoxin:NADP(H) reductase (FNR), but the specific mechanism of FNR-mediated protection remains enigmatic. It has also been reported that the localization of this enzyme within the chloroplast is related to its role in stress tolerance. Here, we dissected the impact of FNR content and location on photoinactivation of photosystem I (PSI) and photosystem II (PSII) during high light stress of Arabidopsis (*Arabidopsis thaliana*)*.* The reaction center of PSII is efficiently turned over during light stress, while damage to PSI takes much longer to repair. Our results indicate a PSI sepcific effect, where efficient oxidation of the PSI primary donor (P700) upon transition from darkness to light, depends on FNR recruitment to the thylakoid membrane tether proteins: thylakoid rhodanase-like protein (TROL) and translocon at the inner envelope of chloroplasts 62 (Tic62). When these interactions were disrupted, PSI photoinactivation occurred. In contrast, there was a moderate delay in the onset of PSII damage. Based on measurements of ΔpH formation and cyclic electron flow, we propose that FNR location influences the speed at which photosynthetic control is induced, resulting in specific impact on PSI damage. Membrane tethering of FNR therefore plays a role in alleviating high light stress, by regulating electron distribution during short-term responses to light.

## Teaser

Altered location of a key enzyme involved in the post-photosystem I electron transport chain ameliorates damage to photosystem I during increasing light intensity.

## Introduction

Electrons excited at photosystem I (PSI) reduce ferredoxin (Fd), and the majority of these are used by the enzyme Fd:NADP(H) oxidoreductase (FNR) to generate NADPH (Shin et al., 1963), which is required for CO_2_ fixation. In Angiosperms, FNR is tightly bound to the thylakoid membrane tethers thylakoid rhodanase-like (TROL) protein and translocon at the inner envelope of chloroplasts 62 (Tic62) in the dark (Juric et al., 2009; Benz et al., 2010; Yang et al., 2016), but this association is weakened at higher pH in vitro, and in the light (Alte et al., 2010). Association of FNR with PSI is also reported (Andersen et al., 1992; Marco et al., 2019), and based on immunogold labeling it was proposed that FNR released from the Tic62/TROL tethers in the presence of light could be localized at PSI (Kramer et al., 2021). Oxidized PSI is re-reduced with electrons from plastocyanin (Hippler and Drepper, 2006), which shuttles them through the thylakoid lumen from the cytochrome b_6_*f* complex (Cyt b_6_*f*). The Cyt b_6_*f* is reduced in turn by plastoquinol (PQH_2_), which is the electron acceptor at photosystem II (PSII). This plastoquinone (PQ) reduction and oxidation drives formation of ΔpH, which is used by the thylakoid ATPase to synthesize ATP.

Photoexcitation of PSI can alternatively be used to form ΔpH by returning electrons from Fd to PQ through at least two cyclic electron flow (CEF) pathways (Munekage et al., 2004). One Fd:PQ reductase is a homolog of respiratory complex I, known as the NDH (Burrows et al., 1998; Shikanai et al., 1998; Yamamoto and Shikanai, 2013), while the mechanism of the other Fd:PQ reductase, which is inhibited by antimycin A (AA), remains enigmatic (Munekage et al., 2002; Fisher and Kramer, 2014; Nawrocki et al., 2019). Proposed mechanisms involve the function of the proton-gradient regulation 5 (Pgr5) and Pgr5 like 1 (PgrL1) proteins (Munekage et al., 2002; Hertle et al., 2013) and/or direct electron donation from Fd or even FNR to PQ at the Cyt b_6_*f* (Zhang et al., 2001; Nawrocki et al., 2019; Sarewicz et al., 2021). FNR has also been co-purified with the Cyt b_6_*f* (Clark et al., 1984; Zhang et al., 2001) and interaction is reported with PgrL1 (DalCorso et al., 2008). We have recently reported that FNR binding to the thylakoid through either the TROL or Tic62 membrane tethers is necessary for CEF when Arabidopsis (*Arabidopsis thaliana*) plants are transferred from the dark to the light (Kramer et al., 2021), a condition in which CEF is upregulated (Joliot and Joliot, 2006).

Production of damaging reactive oxygen species (ROS) increases in the absence of acceptors at either photosystem. If PQ availability at PSII is limiting, excitation is transferred from triplet-state chlorophyll to O_2_, producing dangerous singlet oxygen (^1^O_2_) (Telfer et al., 1994; Krieger-Liszkay, 2005). This occurs even at very low light, and the reaction center subunit of PSII (D1) is constantly turned over to ensure functional PSII (Fufezan et al., 2002). To minimize such damage, excitation at PSII can be dissipated through non-photochemical quenching (NPQ) mechanisms induced by acidification of the thylakoid lumen (Ruban, 2016; Ruban and Wilson, 2020). Upregulation of this thermal dissipation in vivo requires the PsbS protein and de-epoxidation of the xanthophyll pigment violaxanthin into zeaxanthin, both of which are triggered by the formation of ΔpH (Ware et al., 2015; Sacharz et al., 2017; Steen et al., 2020; Saccon et al., 2020a, 2020b).

If oxidized Fd availability at PSI is limiting, excited electrons are passed from the iron–sulfur (FeS) clusters at the stromal side of PSI to oxygen, forming superoxide (O_2_^•−^) (Allen and Hall, 1974), which is rapidly dismutated enzymatically to hydrogen peroxide (H_2_O_2_) (Asada, 1999). This H_2_O_2_ can be converted to hydroxyl radicals (^•^OH) through Fenton chemistry, a process catalyzed by the PSI FeS clusters in their reduced state, resulting in their rapid destruction by the resulting ^•^OH (Sonoike et al., 1995; Sonoike, 2011). Degradation of the PSI reaction center subunits quickly follows (Ivanov et al., 1998; Tjus et al., 1998) and this requires costly reconstruction of the entire photosystem, which can take days (Kudoh and Sonoike, 2002; Lima-Melo et al., 2019a, 2019b). In angiosperms, this is prevented by restricting electron donation to PSI, maintaining the P700 active center and FeS clusters in an oxidized state (Shimakawa and Miyake, 2019; Storti et al., 2020). PSI donor limitation is promoted either through downregulation/damage to PSII (Ivanov et al., 1998; Tikkanen et al., 2014; Chaux et al., 2015), or through “photosynthetic control” in which PQH_2_ oxidation at the Cyt b_6_*f* is limited by high ΔpH (Tikkanen et al., 2015; Colombo et al., 2016). It has been reported that the role of CEF in photoprotection is related to enhanced generation of ΔpH to induce protective mechanisms, rather than providing alternative PSI acceptors (Rantala et al., 2020).

Experiments on transgenic *Nicotiana tabacum* (tobacco), with FNR either over-expressed or knocked down by antisense, showed that FNR abundance corresponded with tolerance to high light and oxidative stress, and considerable damage at PSII was detected when FNR contents were low (Palatnik et al., 2003; Rodriguez et al., 2007). It was hypothesized that FNR could be affording protection through its role as a PSI acceptor, preventing build-up of electrons in the thylakoid and possibly acting as a diaphorase to remove radical species. We previously found that FNR could generate considerable O_2_^•−^ during turnover, and that stress remediation pathways were upregulated in Arabidopsis over-expressing FNR (Kozuleva et al., 2016). We interpreted this as indicating the enzyme might confer stress tolerance by “priming” the plant to induce increased abundance of proteins involved in protective mechanisms, such as superoxide dismutation, prior to stress exposure. FNR:membrane complex associations are also implicated in stress responses. It has been shown that FNR is released from the thylakoid during oxidative stress (Palatnik et al., 1997), and plants with different FNR:membrane associations also vary in stress responses (Lintala et al., 2009, 2012; Kozuleva et al., 2016).

There has been much recent research on PSI photoinhibition, a considerable amount relating to how processes in the post-PSI electron transfer cascade can relieve acceptor limitation and prevent damage (Shimakawa and Miyake, 2018a, 2018b; 2019; Lima-Melo et al., 2019a, 2019b; Leister, 2020; Storti et al., 2020). However, there are no reports of how this might relate to FNR abundance or location, despite the fact that FNR abundance strongly corresponds to stress tolerance (Palatnik et al., 1997, 2003; Rodriguez et al., 2007; Kozuleva et al., 2016). In the work presented here, we dissect the impact of changing FNR content and sub-chloroplast location on PSI and PSII. We find that when plants with perturbed FNR:membrane interactions are exposed to sudden high light, there is specific photoinactivation of PSI. Dark recovery after illumination shows that PSI damage correlates with this inactivation. Surprisingly, over the same timescale, PSII is slightly less photodamaged than in the wild-type (WT) as measured by fluorescence quenching in the dark (qPd). Protection of PSI correlates with CEF capacity, the rate of proton gradient (ΔpH) generation and is AA dependent, but PsbS-mediated NPQ has a minimal effect on PSI photoprotection. It is suggested that protection at PSI may contribute to the stress tolerance conferred by FNR.

## Results

### Estimating PSI photoinactivation and PSII photoinhibition during illumination

In order to differentiate between impact at PSII and PSI during the illumination period, we first established high light treatment protocols. To estimate the onset of damage at PSII, we measured PSII capacity as Y(II) (Schreiber and Klughammer, 2009) and the value of photochemical qPd, which incorporates a comparison between actual and theoretical F_0_ values (Ruban and Murchie, 2012; Ruban, 2017) to identify the early stages of PSII photodamage. We also defined a protocol to estimate PSI capacity during illumination. Although measurement of PSI capacity in plants following dark adaptation (Pm) is well established (Yamori et al., 2016; Shimakawa and Miyake, 2018a, 2018b; Rantala et al., 2020), this is hard to accurately determine during illumination. We followed the protocol of Klughammer and Schreiber (2008), by briefly turning off the actinic light while applying a 10 s far-red (FR) light pulse prior to a saturating flash. Here, we call this transient P700 maximum (tPm) to help differentiate it from the Pm values calculated following prolonged dark adaptation/recovery.

**Figure 1:**
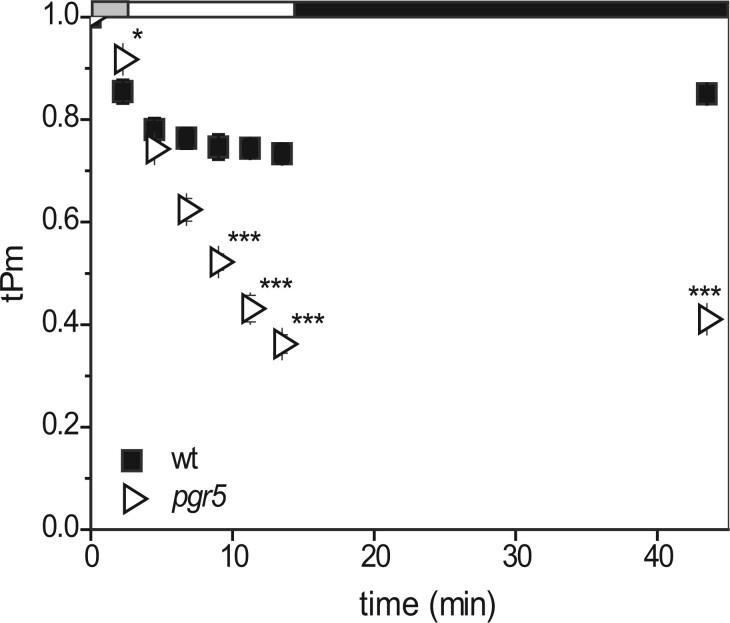
Suitability of the tPm parameter to follow PSI inactivation during the light. Timing of the changes in light intensity is indicated above the graph: The grey bar indicates a step-wise increase to first 543, then 692 µmol photon m^−2^s^−1^ (1 min each). The white bar indicates continuous illumination at 1,385 µmol photon m^−2^s^−1^, and the black bar indicates a dark relaxation period. WT (black squares) and *pgr5* (open right-pointing triangles). tPm was measured upon illumination. Absolute Pm was determined after 30 min of dark adaptation. Data shown are mean ± sem (*n* ≥ 3 individuals per genotype). Differences attributed by post hoc honest significant difference Tukey’s test. *P* values are indicated as “***”  < 0.001, “**”  < 0.01, “*”  < 0.05, “.”  < 0.1.

To examine whether decreases in PSI capacity measured during illumination (tPm) translate into photodamage as calculated by Pm, we compared *A.* *thaliana* WT with a classic genetic model prone to PSI damage, the *pgr5* mutant (Takagi and Miyake, 2018; Rantala et al., 2020; Storti et al., 2020). The tPm is relatively similar in *pgr5* and the WT for the first 5 min of high light (Figure 1), as it drops from 1 to around 0.78. Thereafter, the WT stabilizes at around 0.75, while *pgr5* continues by decreasing further to around 0.35. Some recovery is seen in both genotypes following 30 min in the dark (to 0.85 for the WT and 0.4 for *pgr5*), indicating a component of the decrease in tPm is not related to PSI damage. For this reason, throughout the text, we refer to decreases in tPm as PSI photoinactivation, as it contains a recoverable component, while decreases in Pm following dark relaxation are described as photodamage. A significant ∼0.4 difference between WT and *pgr5* values is consistent between the final tPm and the dark recovered Pm. This confirms the predisposition of *pgr5* to PSI photodamage, and indicates that differences between WT and *pgr5* tPm values are predominantly due to PSI damage. Therefore, at least in these conditions, tPm can provide an estimate of real-time PSI photodamage, and this also confirms that damage to PSI (a decrease in PSI activity by ∼15% after dark recovery) can be achieved in WT Arabidopsis through the application of 15 min of high light.

### FNR abundance and location are related to PSI inactivation

**Figure 2:**
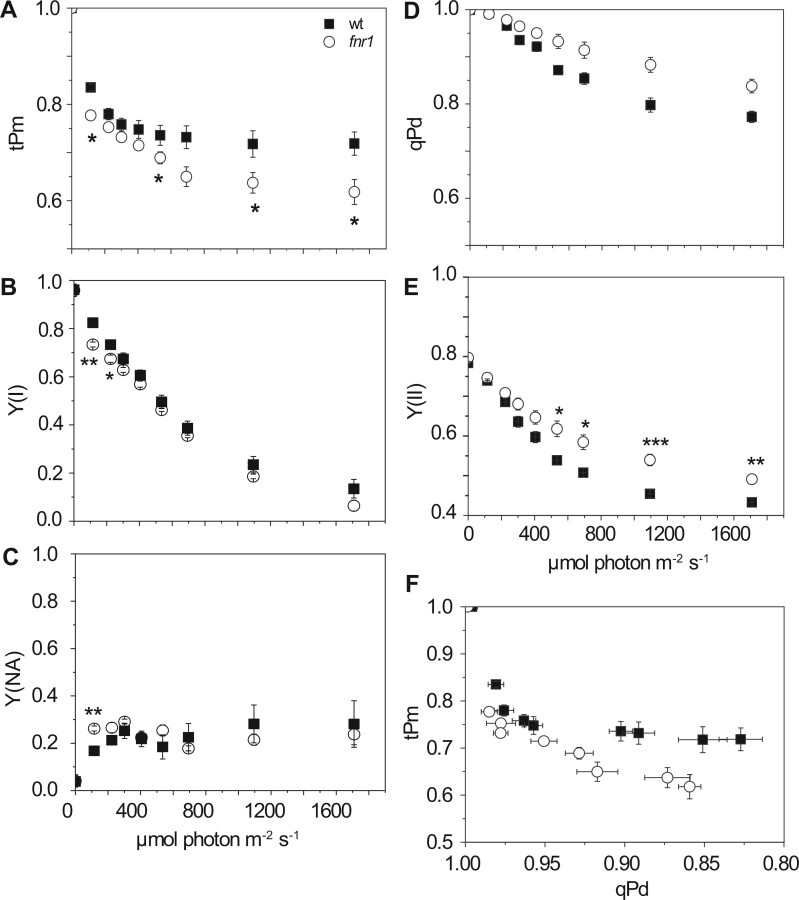
A comparison of PSI and PSII inactivation over increasing light intensity in WT (black squares) and *fnr1* (open circles). A, tPm. B, Effective quantum yield of PSI (Y(I)). C, acceptor limitation at PSI (Y(NA)). D, photochemical quenching of PSII in the dark (qPd). E, Effective quantum yield of PSII (Y(II)). F, Relationship between tPm and qPd. Data shown are mean ± sem (*n* = 5–7 individuals per genotype). Differences attributed by post hoc honest significant difference Tukey’s test. *P* values are indicated as “***”  < 0.001, “**”  < 0.01, “*”  < 0.05, “.”  < 0.1.

As a first step to investigating the relationship between FNR and photoinactivation at PSI and PSII, we analyzed the *fnr1* knockout mutant of FNR1 in Arabidopsis, which has increased susceptibility to high light stress (Kozuleva et al., 2016). This was done over gradually increasing light intensity to allow induction of PSII photoprotective mechanisms (Wilson and Ruban, 2019, 2020). Measurements of tPm (Figure 2A) showed greater photoinactivation of PSI in *fnr1*, both at low and high light intensities. The PSI quantum yield, Y(I), was decreased in *fnr1* relative to the wt at lower light intensities (Figure 2B). As expected for a mutant lacking ∼50% of FNR (a component involved in acceptor regeneration at PSI), the *fnr1* genotype showed significantly greater acceptor limitation, Y(NA), than the WT (Figure 2C). Surprisingly, the *fnr1* genotype shows slightly less photodamage to PSII, as indicated by higher qPd and Y(II) values than WT plants (Figure 2, D and E), although only Y(II) showed statistical significance.

**Figure 3:**
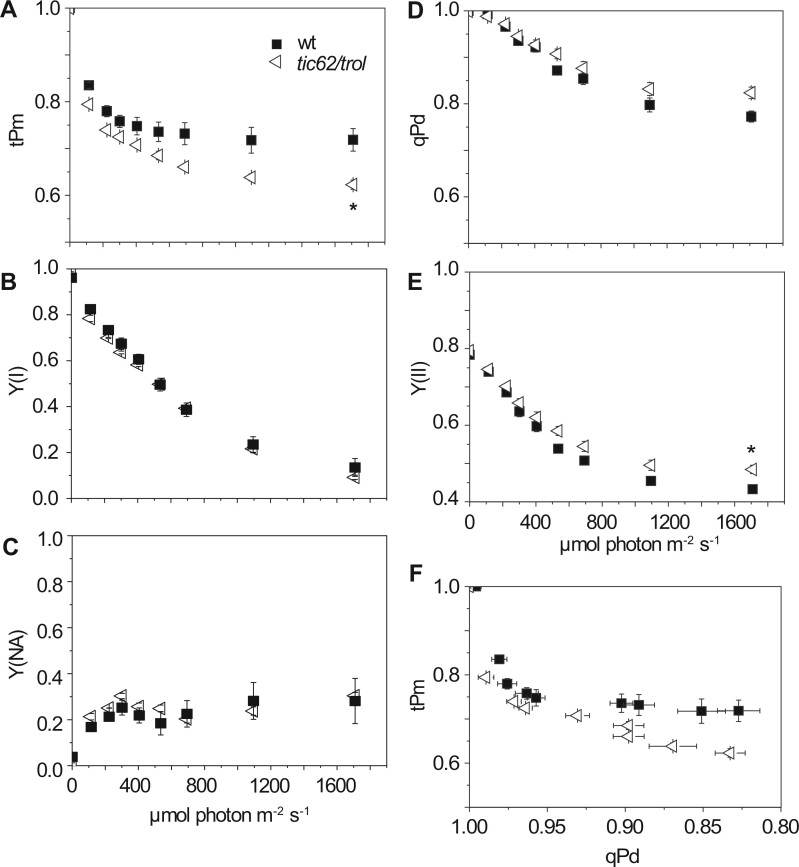
A comparison of PSI and PSII inactivation over increasing light intensity in WT (black squares) and *tic62/trol* (open left-pointing triangles). A, tPm. B, Effective quantum yield of PSI (Y(I)). C, Acceptor limitation at PSI (Y(NA)). D, qPd. E, Effective quantum yield at PSII (Y(II)). F, Relationship between tPm and qPd. Data shown are means ± sem (*n* = 5–7 individuals per genotype). Differences attributed by post hoc honest significant difference Tukey. *P* values indicated by “***”  < 0.001, “**”  < 0.01, “*” < 0.05, “.”  < 0.1.

The *fnr1* plants not only lack around 50% of the WT FNR, but also additionally show a change in the location of the remaining enzyme (Lintala et al., 2007; Hanke et al., 2008), due to low interaction with Tic62 and TROL (Benz et al., 2010). To understand whether the phenotype of *fnr1* is due to decreased FNR content or altered FNR interactions, we analyzed the *tic62/trol* double mutant, which lacks both FNR:membrane tether proteins. The *tic62/trol* mutant retains higher levels of FNR than the *fnr1* mutant, although still less than the WT (Lintala et al., 2014). Relative to the WT, *tic62/trol* and *fnr1* show a similar decrease in tPm (Figure 3A), which becomes significant at higher light intensities, although the PSI acceptor limitation is much lower in *tic62/trol* (Figure 3C). Similar increases in qPd and Y(II) are also seen between the genotypes (Figure 3, D and E). To compare real-time photoinactivation at PSI and photodamage to PSII, we plotted qPd against tPm (Figures 2, F and 3, F). Although there appears to be a negative correlation between tPm and qPd, this is unlikely to be causative, because the parameters show poor correlation in the WT, *fnr1* and *tic62*/*trol* genotypes (*R* ^2^= 0.330, 0.451, and 0.512, respectively).

Both *fnr1* and *tic62/trol* plants have disrupted FNR:tether protein interaction and decreased total FNR content. To try and distinguish the impact of FNR interactions from FNR abundance, we exploited plants in which the *fnr1* mutant was complemented with genes for FNR proteins that specifically interact with TROL (ZmFNR1), Tic62 (ZmFNR2) and only show weak interaction with either tether (ZmFNR3) (Twachtmann et al., 2012; Kramer et al., 2021). Chloroplasts of these plants previously showed equivalent FNR immunostaining to the WT (Kramer et al., 2021). We aimed to use the *fnr1*:Zm*FNR3* line to establish whether WT levels of enzyme, weakly bound to the Tic62/TROL tethers could prevent the decreased tPm seen in *fnr1* and *tic62/trol*. Because antigenicity of iso-proteins may vary, we confirmed that NADPH dependent Fd reduction activity of leaf crude extracts was roughly equivalent between WT (0.26 µmol^−1^ µg protein^−1^ s ± 0.002 sd) and *fnr1*:Zm*FNR3* (0.24 µmol^−1^ µg protein^−1^ s ± 0.014 sd). On increasing light intensities, the PSI parameters of *fnr1*:ZmFNR3 did not fully recover to WT levels, although this was not statistically significant, and developed significantly higher Y(II) values (Supplemental Figure S1). This similar profile of *fnr1*:Zm*FNR3* to *tic62/trol* and *fnr1* indicates that membrane tether association might indeed be related to the relationship between FNR and stress tolerance. By contrast, Supplemental Figure S1 shows that *fnr1*:ZmFNR1 and *fnr1*:ZmFNR2, which interact strongly with TROL and Tic62, respectively, were almost identical to the WT in terms of tPm, Y(I), qPd, and Y(II).

### PSI photoinactivation is partly independent of acceptor limitation

To further probe the relationship between stress tolerance and FNR:membrane tether interactions, we selected three specific genotypes for further experiments: WT; the *tic62/trol* mutant, which lacks FNR:membrane tether interactions; and the *fnr1*:Zm*FNR1* line, which contains WT levels of FNR and increased FNR:TROL interaction (Kramer et al., 2021). Interaction of FNR with TROL is stronger than Tic62 (Alte et al., 2010), so *fnr1*:Zm*FNR1* has exaggerated FNR:tether binding in comparison to the WT.

**Figure 4:**
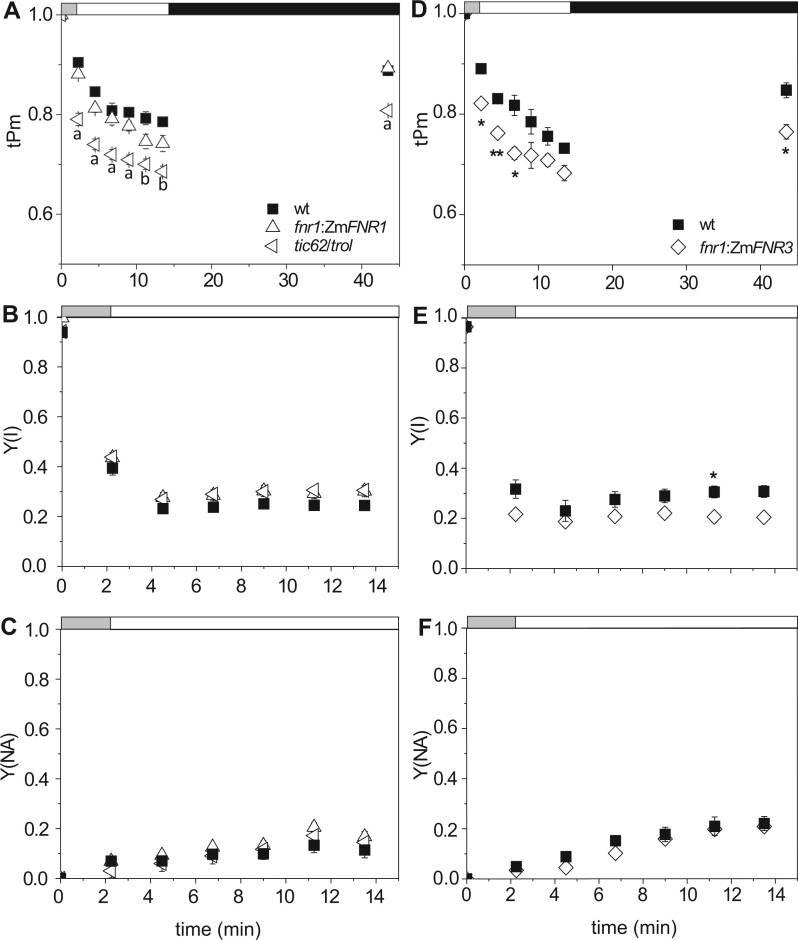
The impact of (FNR:tether interactions on PSI photoinactivation. A–C, Compared 4-week-old WT (black squares), *fnr1*:Zm*FNR1* (open upward-pointing triangles), and *tic62/trol* (open left-pointing triangles) plants. D and E, Compared 6-week-old WT (black squares) and *fnr1*:Zm*FNR3* (open diamonds) plants. Timing of changes in light intensity is indicated above the graph: the gray bar indicates a step-wise increase to first 543, then 692 µmol photon m^−2^s^−1^ (1 min each). The white bar indicates continuous illumination at 1,385 µmol photon m^−2^s^−1^, and the black bar indicates a dark relaxation period. A and D, tPm was measured over illumination. Absolute Pm was determined after 30 min of dark adaptation. B and E, Effective quantum yield of PSI (Y(I)). C and F, Acceptor limitation at PSI (Y(NA)). A–C, Data shown are means ± sem (*n* ≥ 3 individuals per genotype). Significant differences were attributed by post-hoc honestly significant difference Tukey Test and *P* values <0.05 are indicated as the following letters: “a” for WT, *fnr1:*Zm*FNR1 *≠* tic62/trol*; “b” for WT ≠ *tic62/trol*. D–F, Differences from WT attributed by Student’s *t* test. *P* values indicated by “***”<0.001, “**”<0.01, “*”<0.05, “.”<0.1.

These genotypes were then subjected to a sudden application of high light (as in Figure 1) to promote rapid PSI damage*.*Figure 4A shows that tPm values were significantly lower in the *tic62/trol* mutant over the high light treatment (Figure 4A). This is despite the fact that Y(NA) values indicate acceptor limitation does not vary between WT and *tic62/trol* (Figure 4C). In contrast to the *pgr5* mutant (Figure 1), PSI inactivation occurred rapidly during the initial part of the treatment. Importantly, the statistically significant ∼0.1 difference between WT and *tic62/trol* tPm values over illumination is also seen in the Pm values, measured after a 30-min dark recovery. This indicates that the difference between WT and *tic62/trol* tPm values is due to increased photodamage at PSI*.* Although FNR content in the *tic62/trol* mutant is greater than the *fnr1* mutant, it is still decreased in comparison to the WT (Lintala et al., 2014). As a further control to distinguish between the impact of decreased total FNR dose and decreased FNR:Tic62/TROL interactions, we also tested the *fnr1* mutant line complemented with maize FNR3, which has a low affinity for TROL and Tic62. Interestingly, treatment of *fnr1*:Zm*FNR3* with sudden high light resulted in significantly decreased tPm values relative to WT within 1 min (Figure 4D). As in the case of the *tic62/trol* mutant, there was no difference in PSI acceptor limitation as measured by Y(NA) for either *fnr1*:Zm*FNR3* (Figure 4F) or the *fnr1* mutant (Supplemental Figure S2). This confirms that the impact of FNR:tether interactions on PSI photodamage is at least partly independent of acceptor limitation.

### FNR abundance and location are important for CEF and ΔpH generation

**Figure 5:**
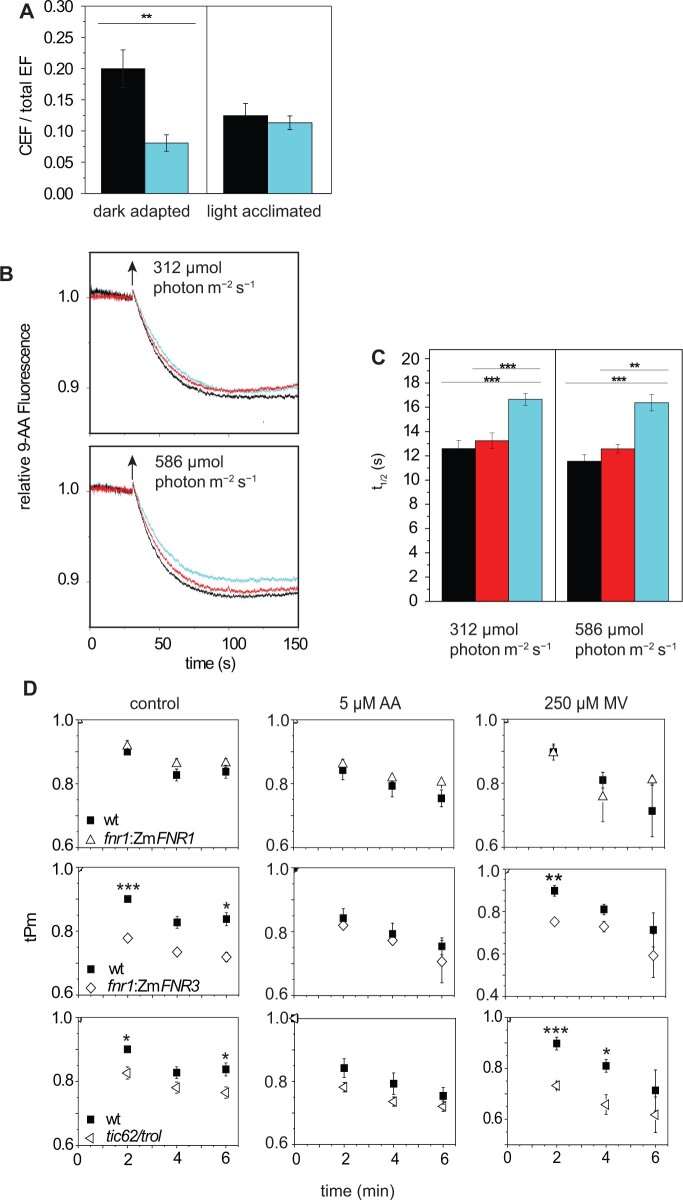
The impact of :FNR:tether interactions on CEF and generation of ΔpH. Genotypes are: WT (black), *fnr1*:Zm*FNR1* (red) and *tic62/trol* (cyan). A, Comparison of the maximum rate of leaf CEF as a function of total electron flow. Values calculated from ECS measurements following a 20 s high light pulse (actinic light for total electron flow, FR light for CEF). Means of measurements on three to five individuals per genotype ± sem. Light acclimation was for 5 min at 150 µmol photons m^−2^s^−1^. B, ΔpH generation by isolated chloroplasts, measured by 9-AA fluorescence quenching. Onset of actinic irradiance is indicated by upward arrow. Irradiance at 312 (upper graph) and 586 µmol photon m^−2^s^−1^ (lower graph). Traces are averages of three independent experiments. C, Rate of ΔpH generation, calculated as *t*_1/2_ on illumination at the indicated light intensities. Values are means ± sem of experiments on three independent chloroplast preparations for each genotype. Differences attributed by post-hoc honestly significant difference Tukey’s test for half times for rate of ΔpH generation and Student *t* test for CEF analysis. *P* values are indicated as “***”<0.001, “**”<0.01, “*”<0.05, “.”<0.1. D, Impact of inhibitors on tPm determination following sudden illumination at 1,095 µmol photon m^−2^ s^−1^. Individual leaves were dark-adapted for 40 min and infiltrated with buffer, 5 µM AA or 250 µM MV, prior to illumination and measurement of tPm at the indicated times. Data are means ± sem (*n*  ≥ 3 individuals per genotype). Differences from WT values attributed by Student’s *t* test. *P* value indicated by “***”<0.001, “**”<0.01, “*”<0.05.

Apart from preventing acceptor limitation at PSI, another mechanism by which FNR could prevent photoinactivation is through a role in CEF, to restrict electron supply to PSI. We have recently shown that short-term induction of CEF on transfer from dark to light is dependent on FNR:tether interactions (Kramer et al., 2021). This data showed that the *fnr1* mutant lost CEF capacity, and that this could be rescued by expression of Zm*FNR1* (strong TROL binding) but not Zm*FNR3* (weak tether interactions). We, therefore, compared relative CEF capacity of WT and the *tic62/trol* mutant following either dark adaptation, or light acclimation (Figure 5A). CEF is elevated in the WT after dark adaptation as previously reported (Kramer et al., 2021), but not in the *tic62/trol* mutant. CEF has the capacity to accelerate generation of ΔpH and we therefore compared the rate at which ΔpH is generated at the onset of light. Initially, 312 µmol photon m^−2^ s^−1^ was used, and we confirmed that this light intensity was saturating by repeating the experiment at 586 µmol photon m^−2^ s^−1^ (Figure 5B). Dark-adapted chloroplast preparations from WT, *fnr1*:Zm-*FNR1*, and *tic62/trol*, had equivalent *F_v_/F_m_* values of 0.74 ± 0.01, 0.72 ± 0.04, and 0.70 ± 0.03, respectively, indicating that chloroplasts were of equivalent quality. We found no significant difference between the amplitude of fluorescence quenching of the three genotypes. However, we observed a ∼30% increase in the half time to reach maximum fluorescence quenching in *tic62/trol* compared to the WT and *fnr1*:Zm*FNR1* (Figure 5C) at both irradiance intensities. As the same genotype shows an inability to upregulate CEF on dark adaptation, caused by disruption of FNR:tether interactions, it is highly likely that the decreased rate of ΔpH development upon transitions from darkness to light is due to a missing contribution of CEF to proton pumping. To further investigate whether an inability to upregulate CEF on transition to higher light results in the decreased tPm values measured in the *tic62/trol* and *fnr1*:Zm*FNR3* plants, we examined the impact of the classical CEF inhibitor AA on tPm. Figure 5D shows that infiltration of leaves with 5 µM AA is sufficient to eliminate the difference in tPm between WT and *fnr1*:Zm*FNR3*, while the difference between WT and *tic62/trol* ceases to be significant. Infiltration with methyl viologen (MV), which should compete with FNR for reduced electrons, also eliminates this difference but only on a longer timescale.

**Figure 6:**
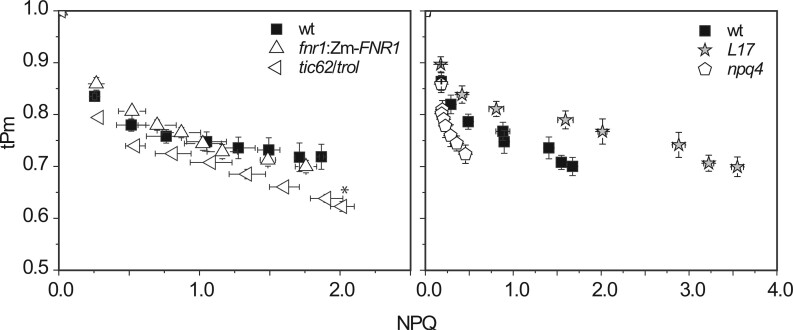
Examining a role for NPQ in FNR dependent protection of PSI. Increasing actinic light intensity was applied to plants and tPm and NPQ parameters calculated as described in “Materials and methods”. Genotypes in the left panel are WT (black squares), *fnr1*:Zm*FNR1* (open upward-pointing triangles), *tic62/trol* (open left-pointing triangles). Genotypes in the right panel are *L17* (gray stars) and *npq4* (open pentagons). Data shown are means ± sem (*n* ≥ 3 individuals per genotype). Significant differences were attributed by post-hoc honestly significant difference Tukey’s test. Only tPm differences are indicated on this Figure: *P* values are indicated as “***”<0.001, “**”<0.01, “*”<0.05, “.”<0.1. See Supplemental Figure S3 for original NPQ and tPm values plotted against light intensity.

Faster development of ΔpH might accelerate PSI oxidation, and therefore protection, by inducing PsbS-dependent NPQ to limit the flux of electrons from PSII (Tikkanen et al., 2014; Chaux et al., 2015). We, therefore, compared how NPQ induction over increasing light intensities influenced inactivation of PSI (tPm). Figure 6 shows that there is no significant difference in NPQ values between the genotypes, and that tPm does not correlate with NPQ development over increasing light intensity. To further evaluate whether PsbS-dependent NPQ might play a role in protecting PSI from photoinactivation in Arabidopsis, we also measured genotypes with enhanced NPQ (*L17*) (Li et al., 2002), or compromised NPQ (*npq4*) (Havaux and Niyogi, 1999; Figure 6), confirming that, under these conditions at least, there is no relationship between NPQ and tPm*.* We further treated these genotypes with the sudden high light protocol (Supplemental Figure S3, C–E) which resulted in similar tPm and final Pm values for WT, *L17*, and *npq4*. This result suggests that NPQ does not limit PSI re-reduction in either condition measured here.

**Figure 7:**
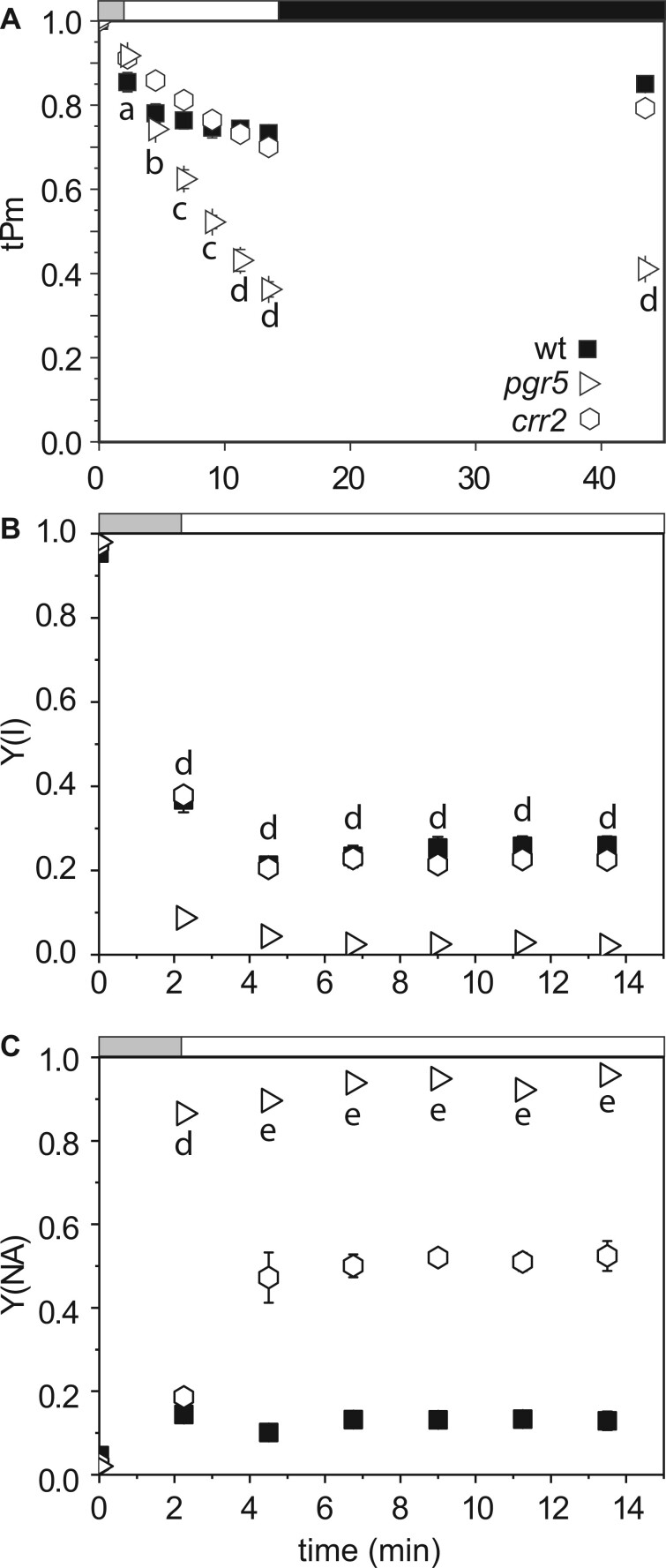
Examining the contribution of NDH and pgr5 mediated CEF to tPm protection in high light stress. P700 absorption and chlorophyll *a* fluorescence was assessed on *A. thaliana* genotypes: WT (black squares), *pgr5* (open right-pointing triangles) and *crr2* (open hexagons). Timing of changes in light intensity is indicated above the graph: the gray bar indicates a step-wise increase to first 543, then 692 µmol photon m^−2^s^−1^ (1 min each), the white bar indicates continuous illumination at 1,385 µmol photon m^−2^s^−1^, and the black bar indicates a dark relaxation period. A, tPm was measured over illumination and absolute Pm was determined after 30 min of dark adaptation. B, Effective quantum yield of PSI (Y(I)). C, acceptor limitation at PSI (Y(NA)). Data shown are means ± sem (*n* ≥ 3 individuals per genotype). Significant differences were attributed by post-hoc honestly significant difference Tukey’s test and *P* values under 0.05 are indicated by are indicated as the following letters: “a” for *pgr5* and *crr2 *≠* *WT; “b” for *pgr5* and WT ≠ *crr2*; “c” for *pgr5* ≠* * *crr2*; “d” for *pgr5* ≠ WT and *crr2*; “e” for *pgr5 *≠* *WT.

Any mechanistic role of FNR in CEF remains unclear. To compare the impact of FNR:*Tic62*/*TROL* interaction with the classical pathways of CEF, we used the same protocol to check whether a similar response could be measured in *pgr5* (a mutant of the Pgr5/PgrL1 pathway, as shown in Figure 1) and *crr2* (an assembly factor mutant of the NDH complex). In Figure 7, neither of these mutants show a similar pattern of PSI inactivation to *tic62/trol, fnr1*, or *fnr1*:Zm*FNR3* plants. On sudden high light illumination of *pgr5*, tPm is initially higher than in WT and then gradually decreases over the time course, to much lower values than other genotypes (Figure 7A), while *crr2* also shows higher initial tPm values than WT, but does not decrease below WT values. Y(NA) values indicate increased acceptor limitation relative to wt for both *pgr5* and *crr2* (Figure 7C). Interestingly, when these genotypes are subject to an increasing light protocol (Supplemental Figure S4) *crr2* plants present higher tPm values than the WT up to light intensities of 1,096 µmol photons m^−2^ s^−1^.

### FNR impact on PSI photoinactivation upon fluctuating light

**Figure 8:**
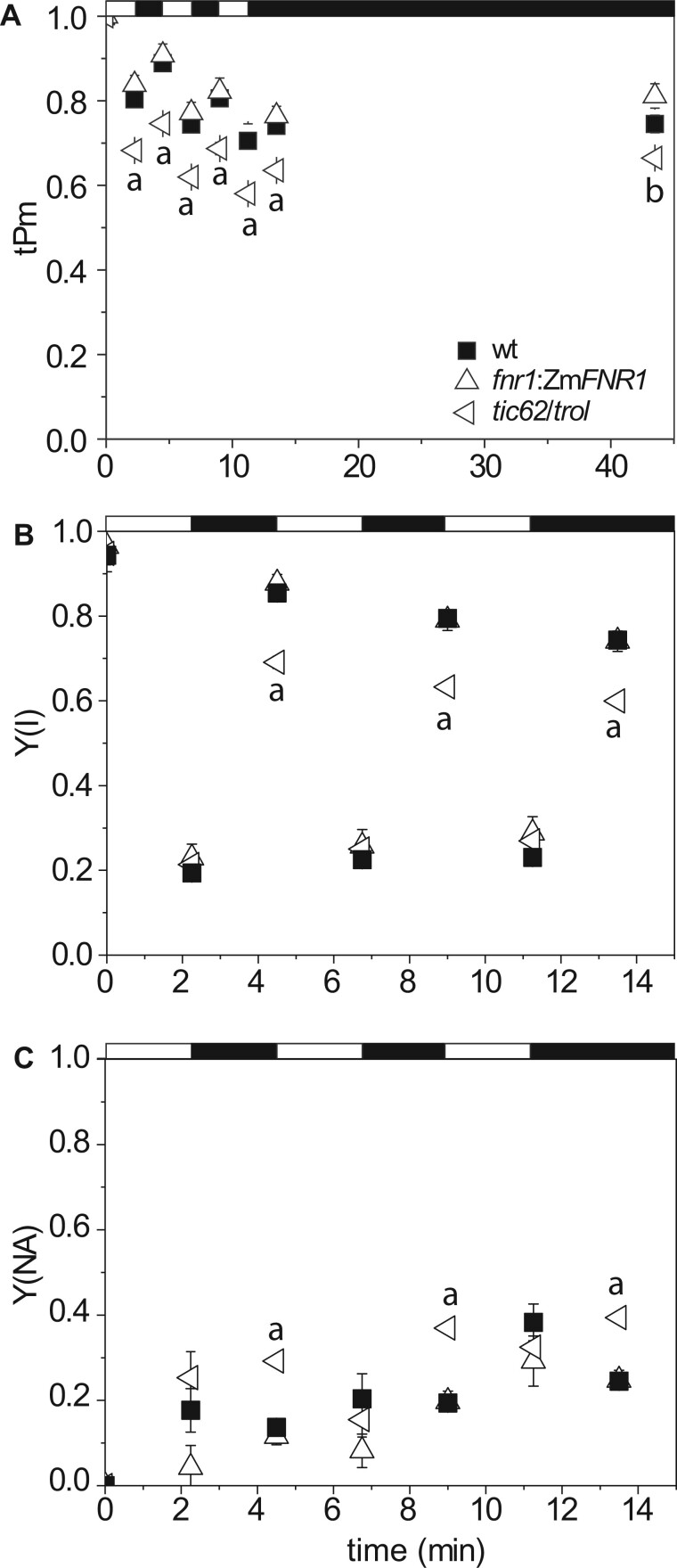
Impact of FNR:tether interactions on PSI inactivation during short-term light fluctuation. Timing of changes in light intensity is indicated above the graphs: black bars = darkness, while white bars = 1,385 µmol photon m^−2^s^−1^. P700 absorption and chlorophyll *a* fluorescence were followed in *A. thaliana* genotypes: WT (black squares), *fnr1*:Zm*-FNR1* (open upward-pointing triangles), and *tic62/trol* (open left-pointing triangles). A, tPm with absolute Pm determined after 30 min of dark adaptation following light treatment. B, Effective quantum yield of PSI (Y(I)). C, acceptor limitation at PSI (Y(NA)). Data shown are means ± sem (*n* ≥ 3 individuals per genotype). Significant differences were attributed by post-hoc honestly significant difference Tukey’s test and are indicated by the following letters: “a” for *fnr1*:Zm*-FNR1* and WT ≠ *tic62/trol*; “b” for *fnr1*:Zm*-FNR1 *≠* tic62/trol*.

It has been frequently reported that fluctuating light exacerbates the damage to PSI caused by perturbation of CEF (Suorsa et al., 2012, 2013; Kono et al., 2014; Huang et al., 2018; Nikkanen et al., 2018), and we therefore repeated our experiment using a fluctuating light environment. This comprised three phases of high light (1385 μmol photon m^−2^ s^−1^) intensity illumination alternating with total darkness (Figure 8). Following the first light treatment, tPm values were lower for the *tic62/trol* mutant throughout the illumination protocol (Figure 8A). When measured during illumination periods, genotype had no effect on PSI yield and acceptor limitation (Figure 8B, Figure 8C). There were significantly lower Y(I) and higher Y(NA) values for *tic62/trol* following the dark steps. The final Pm values were lower after this fluctuating light routine (Figure 8A) relative to those obtained after continuous high light (Figure 4A) in all the genotypes. Interestingly, the dark recovery of *Pm* only decreased by ∼9% in *fnr1*:Zm-*FNR1* which, unlike the WT, retained a significant difference from *tic62/trol*.

## Discussion

We hypothesized that, as FNR is a critical component of the post-PSI electron transfer cascade, its abundance, and/or location on the membrane (close to PSI), might contribute to PSI protection. This could be analogous to the impact of the flavodiiron proteins (Shimakawa et al., 2017; Alboresi et al., 2019), or photorespiration (Allahverdiyeva et al., 2011; Hanawa et al., 2017; Takagi et al., 2017), providing excess sinks to maintain P700 in a protected, oxidized state. Because differences in electron transport induced by altered FNR content or location are most pronounced in the early stages of illumination following dark adaptation (Twachtmann et al., 2012; Kramer et al., 2021), it was critical to estimate PSI inactivation during the early stages of illumination, for which we used the tPm parameter (Figure 1). This revealed that, over the relatively short high light stress applied here, FNR content and membrane association specifically impact on photoinactivation of PSI. Interestingly, the increased PSI photoinactivation in *fnr1*, *fnr1*:Zm*FNR3*, and *tic62/trol* mutants occurs at the same time as a small, but significant improvement in PSII protection.

PSI acceptor limitation is associated with O_2_^•−^ generation (Takahashi and Asada, 1988; Kozuleva et al., 2014), and FNR can also generate O_2_^•−^ during catalysis (Kozuleva et al., 2016). Perhaps surprisingly, detection of light-dependent free radical evolution in antisense tobacco leaves indicated that decreased FNR content resulted in no difference in O_2_^•−^, but rather an increase in ^1^O_2_ (Palatnik et al., 2003), a radical normally associated with PSII damage. In addition, spin-trapping of O_2_^•−^ produced by chloroplasts from Arabidopsis single *trol* knockout plants was previously shown to be lower than in WT in both light and dark (Vojta et al., 2015). The results in tobacco prompted the suggestion that PSII was acceptor limited, due to back up of electrons through the whole electron transport chain (Palatnik et al., 2003). This might not be contradictory to our data, as the light stress applied to tobacco FNR antisense plants prior to measurement was for much longer time scales than used in our study. Our data (Figures 2–4 and 8;Supplemental Figures S1 and S2) indicate that the FNR associated PSI photoinactivation occurs early in illumination. In the absence of correct FNR:tether interaction, poor initial regulation of events around PSI on illumination, or an increase in light intensity, may therefore cause a temporary increase in O_2_^•−^ (and therefore ^•^OH) production relative to the WT. PSI damage would then progress from the destruction of FeS clusters to protein subunits at a small percentage of PSI complexes, resulting in ^1^O_2_ production from triplet state chlorophyll at damaged PSI (Sonoike et al., 1995; Tjus et al., 1998). PSII is highly sensitive to ^1^O_2_ (Krieger-Liszkay, 2005) and so a small amount of PSI damage could result in considerable PSII damage (Sonoike, 2011). The small, short-term improvement in PSII tolerance to high light stress seen in plants with disrupted FNR:tether interactions might have a number of causes. These include secondary effects to decrease light harvesting or increase PQ acceptor availability at PSII in response to the direct impact at PSI, and are beyond the scope of this study.

We expected that the main impact of FNR abundance and/or location on PSI damage would relate to acceptor limitation. However, our results indicate that the effect is more complex. Although acceptor limitation (as measured by Y(NA)) is elevated on initial increases in light intensities for the *fnr1* mutant (Figure 2), this is not the case for the *tic62/trol* mutant (Figure 3, Supplemental Figure S1) neither for *tic62/trol* or *fnr1:ZmFNR3* on sudden high light (Figure 4). Moreover, infiltration of leaves prior to measurement with MV, which eliminates acceptor limitation at PSI, does not immediately diminish the difference in tPm between these genotypes and the WT (Figure 5), suggesting that acceptor limitation is not the predominant cause of their increased PSI photoinactivation. The inhibition by MV later in the time course presumably arises from superoxide production, and it is interesting that it does not immediately impact on whatever protective function FNR is performing in sudden high light. This may be related to reports that MV is actually a relatively weak inhibitor of electron transfer by Fd between PSI and FNR (Setif, 2015), meaning that under the conditions used here sufficient reduced Fd is probably still available to FNR.

The increasing light experiments (Figures 2 and 3) were designed to distinguish damage at PSII from PSI in conditions where NPQ was able to develop, while the sudden light increase (Figure 4) tests PSI activity when electron flux from PSII is maximum. The stronger binding of FNR to the Tic62/TROL tether proteins in WT and *fnr1*:Zm*FNR1* lines at the onset of the high light period is therefore a more likely explanation for their higher tPm values than in *fnr1, tic62/trol* or *fnr1:*Zm*FNR3*, rather than differences in FNR abundance. The data indicate that, without NPQ, PSI acceptors are overwhelmed irrespective of any differences in FNR content seen between our genotypes. We included the *fnr1*:Zm*FNR1* plants in our study to examine whether tighter binding to membrane tethers might enhance PSI protection. In most experiments, this was not the case, but intriguingly *fnr1*:Zm*FNR1* did show slightly higher Pm values than the WT following dark recovery from the fluctuating light protocol (Figure 8).

As we previously reported an impact of FNR location on CEF during the dark to light transition for the *fnr1* and *fnr1*:Zm*FNR3* lines (in which FNR:membrane tether interactions are disrupted) (Kramer et al., 2021), we confirmed that this phenotype was also present in *tic62/trol* mutants (Figure 5A). The absence of this CEF might be expected to result in slower generation of ΔpH, which is indeed the case for this genotype (Figure 5B). We could not detect a significant difference in steady-state ΔpH, which might be expected as it is subject to extensive regulation (Tikkanen et al., 2015; Colombo et al., 2016; Yang et al., 2018). Many chloroplast protective mechanisms are dependent on ΔpH, including NPQ (Johnson and Ruban, 2011) and photosynthetic control (Colombo et al., 2016). We found no evidence for a relationship between NPQ and tPm or Y(I) in our genotypes (Figure 7;Supplemental Figure S3) and plants with enhanced or attenuated NPQ also showed minimum impact on PSI photoinactivation. This confirms previous reports that PSII downregulation has little effect on PSI protection (Tikkanen et al., 2015; Shimakawa and Miyake, 2018a, 2018b).

An inability to regulate electron donor supply by photosynthetic control has been proposed to cause the extreme PSI damage reported in *pgr5* mutants (Shimakawa and Miyake, 2018a, 2018b; Lima-Melo et al., 2019a, 2019b) and confirmed here (Figures 1 and 7). In this work, we did not compare the dark-adapted and light acclimated CEF capacity of *pgr5* and *crr2* plants, but the same technique has previously been used to show that, while NDH mutants are relatively unaffected, dark-adapted Arabidopsis *pgr5* mutants have decreased total CEF capacity, as measured following 120 s illumination (Suorsa et al., 2016). As the *tic62/trol* plants show lower rates of initial ΔpH generation (Figure 5, B and C), it seems likely that a slower induction of photosynthetic control might also be responsible for the PSI inactivation associated with decreased FNR content and membrane tether interaction.

Interestingly, during the first 5 min of high light stress, the tPm values for *pgr5* plants are higher than or equivalent to WT (Figure 1), and only decrease drastically thereafter. Those of *tic62/trol and fnr1*:Zm*FNR3* (Figure 4), and *fnr1*

(Supplemental Figure S2) show the opposite trend, with an immediate drop relative to WT, after which the relative difference between the genotypes changes little. This is consistent with disruption of a process in the initial stages of illumination, such as the slower induction of ΔpH seen in Figure 5, B and C. FNR has been found in complex with PGR5 in algae (Iwai et al., 2010) and angiosperm PGRL1 was also reported to interact with FNR (DalCorso et al., 2008). AA is the canonical inhibitor of FQR-dependent CEF, and evidence supports a role for PGR5 in its mechanism of action (Sugimoto et al., 2013), while AA also abolishes FNR-dependent differences in tPm (Figure 5D). Although our data do not rule out these proteins acting in concert, they suggest that the mechanism disrupting CEF induction during the dark to light transition is different between the *pgr5* mutant and the *fnr1*, *tic62/trol*, or *fnr1*:Zm*FNR3* genotypes.

One possible explanation for our data is therefore that, in addition to acceptor limitation, the stress phenotype associated with decreased FNR content and tether association could be due to increased donor arrival through slower induction of photosynthetic control. This would explain the specific, short-term inactivation of PSI seen in the *fnr1* and *tic62/trol* genotypes on exposure to high light.

## Materials and methods

### Plant material and growth conditions

Unless otherwise indicated, plants were grown in 12 h light at 21°C, 12 h dark at 18°C, and in 0.12 L pots on a 6:6:1 ratio of John Innes No. 3 soil, Levington M3 potting compost and perlite (Scotts UK, Ipswich, UK). Arabidopsis (*A.* *thaliana*) genotypes ecotype Columbia (WT) and *fnr1* were as described in (Hanke et al., 2008), Arabidopsis plants expressing *Zea mays FNR1, FNR2*, and *FNR3* in the *fnr1* background were as described previously (Kramer et al., 2021) and *tic62/trol* (Lintala et al., 2014) was a kind gift of Paula Mulo. *pgr5* (Munekage et al., 2002)) and *crr2* (Hashimoto et al., 2003) were a kind gift from Toshiharu Shikanai. The *npq4* (Havaux and Niyogi, 1999) and *L17* (Li et al., 2002) were also used in this work. Unless stated otherwise all measurements were carried out on 6-week-old plants that showed no signs of inflorescence or visible anthocyanin accumulation. Plants were dark-adapted for 45 min before each measurement and *F_v_/F_m_* was on average 0.78 ± 0.002 with no significant difference between genotypes.

### Chlorophyll fluorescence and P700 absorption measurements and parameters

Chlorophyll *a* fluorescence and P700 oxidation measurements were performed simultaneously using a DUAL-KLAS-NIR-PAM (Walz, Effeltrich, Germany). In this method, the P700 redox state is monitored by deconvoluting the four absorption signals by differential model plots (Klughammer and Schreiber, 2008).

An assessment of the true quantity of photoinactivated PSII, as photochemical qPd, was calculated as described by Ruban and Murchie (2012).

$$\text{qPd}=\left(\text{Fm}\prime -\text{Fo}\prime_{\text{act}}.\right)/\left(\text{Fm}\prime -\text{Fo}\prime_{\text{calc}}.\right)$$

where *Fm*′ is the maximum fluorescence yield after exposure to actinic light, Fo′_act._ and Fo′_calc._ are the actual and theoretical minimum fluorescence yields measured in the dark after actinic light treatment, respectively. Fo′_calc._ is determined according to the equation of Oxborough and Baker (1997), as defined below.

$$\text{Fo}\prime_{\text{calc}}.=1/\left(1/\text{Fo}-1/\text{Fm}+1/\text{Fm}\prime \right)$$

At lower actinic light levels, Fo′_act._ ≈ Fo′_calc._ and qPd = 1. However, as the intensity of actinic light rises, Fo′_act._ also increases, which is an indicator of photoinactivation. The divergence between Fo′_act._ and Fo′_calc._ is due to permanent closure of RCs and causes qPd < 1.

The effective ‘quantum yield’ of PSII (Y(II) is undermined by two processes induced by excessive light: NPQ and photoinactivation (qPd). The effect of each upon Y(II) was therefore calculated as

Y(II)=(qPd⋅Fv/Fm)/[1+(1−Fv/Fm)⋅NPQ]

Fv/Fm represents the maximum Y(II), which is calculated as (Fm − Fo) / Fm, with Fo and Fm being the minimum and maximum fluorescence yields, respectively. The theoretical Y(II) for a given NPQ is calculated with maximal values of Fv/Fm and qPd as constants. Damage to PSI is usually quantified as a change in the maximum photo-oxidisible fraction of PSI (Pm), as measured following dark adaptation. According to Klughammer and Schreiber (2008), Pm can be determined during illumination by (1) measuring the maximal signal level induced by a saturating pulse (oxidisable P700), and (2) measuring the signal level in the dark several hundred milliseconds after the saturating pulse (baseline level; P700 fully reduced). In this work, we have combined these approaches to estimate activity of PSI during illumination. Actinic light is turned off, and far-red light is applied for 10 s before measurement of Pm with a saturating pulse. To avoid confusion, we refer to measurements following dark adaptation/recovery as Pm (photodamage), while values taken during illumination, from which PSI capacity partially recovers (Figure 1), are termed “transient Pm” or tPm, and reflect steady-state inactivation. Both Pm and tPm measurements were normalized to an initial Pm measured following 45-min dark adaptation prior to illumination. Y(I) and Y(NA) were calculated from the P700 traces according to published protocols (Klughammer and Schreiber, 2008). Typical WT chlorophyll fluorescence and P700 traces are shown in Supplemental Figure S5. For vacuum infiltration experiments, leaves of dark-adapted Arabidopsis plants were individually vacuum infiltrated by placing a whole leaf inside a syringe barrel containing 20 mL of buffer (330 mM sorbitol, 20 mM HEPES, pH 7) with or without AA (5 µM) or MV (250 µM). The narrow syringe opening was blocked and the plunger was used to gently draw air out of the tissue until the leaf lost buoyancy, at which point it was immediately measured.

### Chloroplast extraction and proton gradient determination

The protocol was performed basically as described previously (Saccon et al., 2020a, 2020b). Briefly, Protoplasts were obtained from the mesophyll layer of dark-adapted leaves from Arabidopsis adult plants. To expose the chloroplast-enriched leaf layer to the enzyme solution, the lower epidermis was removed with adhesive tape. Leaves were then floated for 1 h on a solution containing 0.4 M Mannitol, 20 mM KCl, 20 mM MES, 10 mM CaCl_2_, 0.1% bovine serum albumin (BSA) (pH 5.5) in the presence of 1.5% cellulase Onozuka^TM^ R-10 and 0.4% Macerozyme R-10 (Yakult, from Serva, Heidelberg, Germany). The solution was then filtered through a layer of muslin cloth and centrifuged twice (3 min, 100 rcf, 4 C). The obtained protoplasts were resuspended in reaction buffer containing 0.5 sorbitol, 20 mM HEPES, 20 mM MES, 20 mM Na-citrate, 10 mM NaHCO_3_, 15 mM MgCl_2_, 0.1% BSA (pH 8). Their intactness was checked with a bright-field optical microscope and Fv/Fm average values were confirmed.

ΔpH was determined from the measurement of 9-aminoacridine (9-AA) fluorescence using the Dual-ENADPH and Dual-DNADPH modules for the Dual-PAM-100 fluorimeter (Walz, Effeltrich, Germany). Intact chloroplasts (35 μM chlorophyll) were suspended in reaction buffer (as above) in the presence of 5 μM 9-AA. The chloroplasts were treated using 312 or 586 μmol photon m^−2^ s^−1^ actinic light for 5 min, followed by 5 min of darkness. The 9-AA quenching traces were normalized to (fluorescence before illumination – fluorescence after illumination)/fluorescence before illumination. Half time of ΔpH induction (*t*_1/2_) was taken as the time for the fluorescence level to reach half of the maximum value of quenched fluorescence upon illumination.

### CEF measurements

Measurements were performed basically as described previously (Kramer et al., 2021). In brief, plants were dark incubated for 30 min before transfer into actinic light at 150 μmol photon m^−2^ s^−1^. After 20 s and 5 min of illumination, LEF and CEF were measured by following the relaxation kinetics of the carotenoid electrochromic bandshift at 520 nm (corrected with the bandshift at 546 nm). Saturating FR light (λ > 720 nm), was used to fully excite PSI with minimum excitation of PSII and calculate the CEF only signal. Electron flow was estimated from the amplitude of the electrochromic shift (ECS) signal upon excitation with a saturating single turnover flash (5 ns laser pulse). Total electron flow was measured with an actinic flash of 1,100 μmol photon m^−2^ s^−1^ while CEF was measured with only FR light at the maximum setting (estimated as 1,400 μmol photon m^−2^ s^−1^ by the manufacturer). LEF was calculated by subtraction of CEF from total electron flow.

### FNR activity

Crude leaf extracts were prepared from 6-week-old plants by grinding with sand at 4°C in 50 mM Tris–HCl pH 7.5, 100 mM NaCl, 2 mM MgCl_2_, 1 mM pefabloc, 0.1% w/v polyvinylpolypyrrolidone. The supernatant from a 2 min, 2,000 *g* centrifugation at 4°C was taken. FNR in this sample was solubilized by the addition of Triton X-100 to 1% (v/v), before further centrifugation at 15,000 *g* at 4°C for 5 min. FNR activity of this supernatant, equivalent to 30 µg total protein, was measured in the dark using a cytochrome *c* reduction assay in the presence of 20 µM Arabidopsis Fd 2 and an NADPH regeneration system as described previously (Hanke et al., 2005).

### Statistics

The *t* test Student’s, analysis of variance, and post-hoc analysis Tukey’s tests were also performed in R version 3.5.3 (R_Core_Team, 2019).

### Accession numbers

Sequence data from this article can be found in The Arabidopsis Information Resource database (https://www.arabidopsis.org/) or GenBank/EMBL databases under the following accession numbers: maize *FNR1*, BAA88236; maize *FNR2*, BAA88237; maize *FNR3*, ACF85815; *Arabidopsis FNR1*, AT5G66190; *Arabidopsis FNR2*, AT1G20020; maize *Tic62*, ACG28394.1; *Arabidopsis Tic62*, AT3G18890; maize *TROL*, ACF79627.1; *Arabidopsis TROL*, AT4G01050.1; *Arabidopsis* PsbS AT1G44575; *Arabidopsis* pgr5 AT2G05620; *Arabidopsis* PgrL1 AT4G11960; *Arabidopsis* crr2 AT3G46790.

## Supplemental data 

The following materials are available in the online version of this article.

**Supplemental Figure S1.** The impact of FNR:tether interaction on PSI and PSII photoinactivation during increasing light intensity.

**Supplemental Figure S2.** Additional experiments on the impact of FNR:tether associations on PSI and PSII photoinactivation during high light exposure in Arabidopsis.

**Supplemental Figure S3.** Impact of altered NPQ on PSI inactivation over increasing light (A and B) and during sudden high light stress (C–E) in Arabidopsis.

**Supplemental Figure S4.** Impact of two different CEF pathways on photoinactivation of PSI and PSII during a gradual increase of light intensity.

**Supplemental Figure S5.** Example traces of the initial section of a typical PSII chlorophyll fluorescence (red trace) and P700 absorption (blue trace) simultaneous measurement.

## Supplementary Material

kiab550_Supplementary_Data

## Notes

G.T.H., A.V.R., and M.R.H. conceived the original screening and research plans; G.T.H. and A.V.R. supervised the experiments; M.R.H, F.S., and G.F. performed the experiments; S.W. and G.F. provided technical assistance to M.R.H.; M.R.H., and G.T.H. designed the experiments and analyzed the data; G.T.H. conceived the project and both, M.R.H and G.T.H., wrote the article with contributions of all the authors; A.V.R. supervised and completed the writing. G.T.H. agrees to serve as the author responsible for contact and ensures communication.

The author responsible for distribution of materials integral to the findings presented in this article in accordance with the policy described in the Instructions for Authors (https://academic.oup.com/plphys/pages/general-instructions) is: Guy T Hanke (g.hanke@qmul.ac.uk).
