# Supplementary material for: Protection of photosystem I during sudden light stress depends on ferredoxin:NADP(H) reductase abundance and interactions
Source: Plant Physiol. 2021 Nov 22;188(2):1028–42. doi: 10.1093/plphys/kiab550 (PMC8825262; doi:10.1093/plphys/kiab550)
Supplement: kiab550_Supplementary_Data [file kiab550_supplementary_data.pdf]

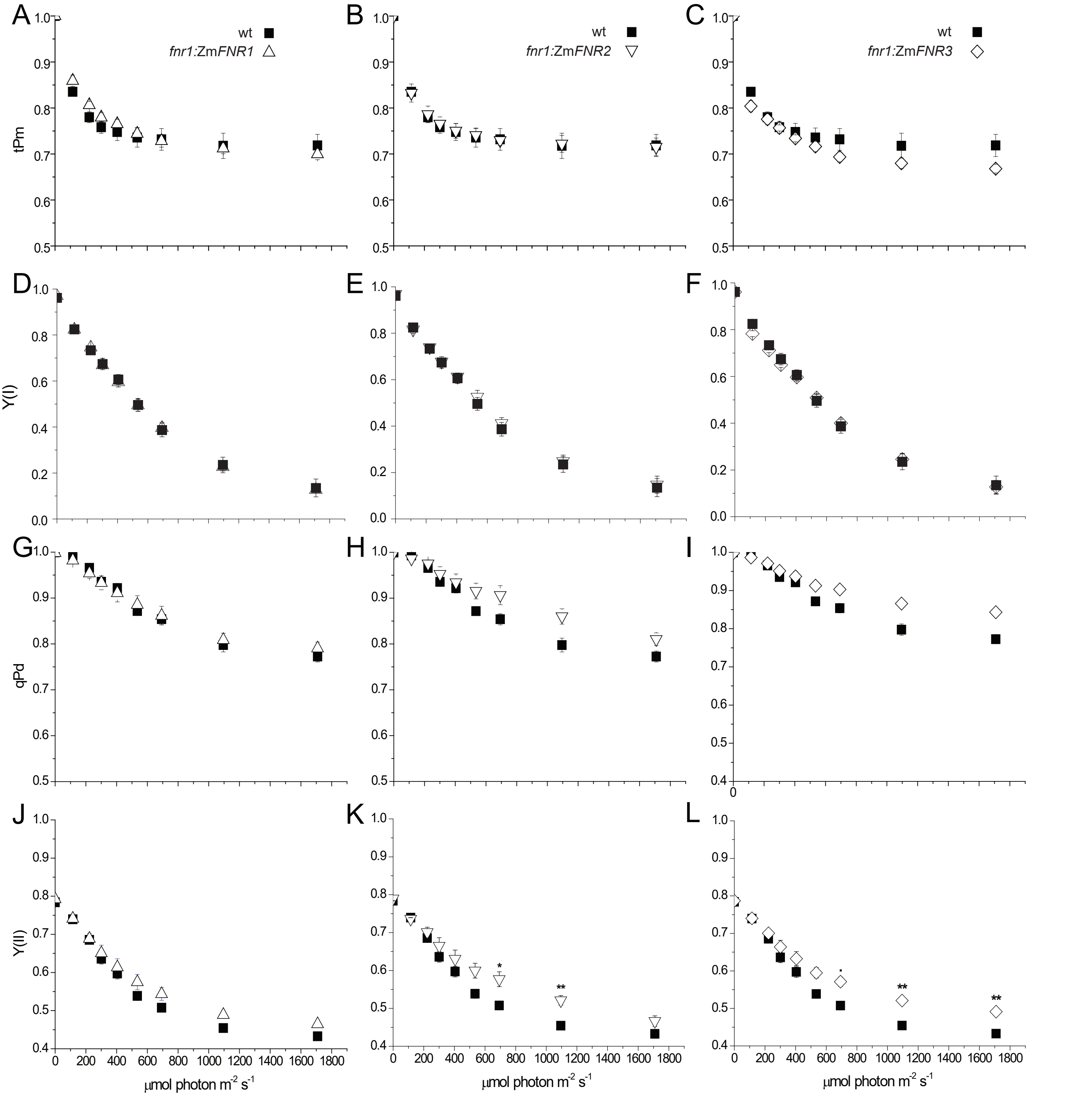

Supplemental Figure S1 The impact of ferredoxin:NADP(H) oxidoreductase (FNR) - membrane tether interaction on photosystem I (PSI) and photosystem II (PSII) photoinactivation during increasing light intensity. Comparison of wt with *fnr1*:Zm*FNR1* (A, D, G, J). Comparison of wt with *fnr1*:Zm*FNR2* (B, E, H, K) and comparison of wt with *fnr1*:Zm*FNR3* (C, F, I, L). A, B, C, transient P700 maximum (tPm). D, E, F, effective quantum yield of PSI (Y(I)). G, H, I, photochemical quenching in the dark (qPd). J, K, L, effective quantum yield of PSII (Y(II)). Data are means ± S.E.M. (n≥3 individuals per genotype). Differences attributed by Student's T-test. P-value indicated by '***'< 0.001, '**'< 0.01, '*'< 0.05, '.'< 0.1.

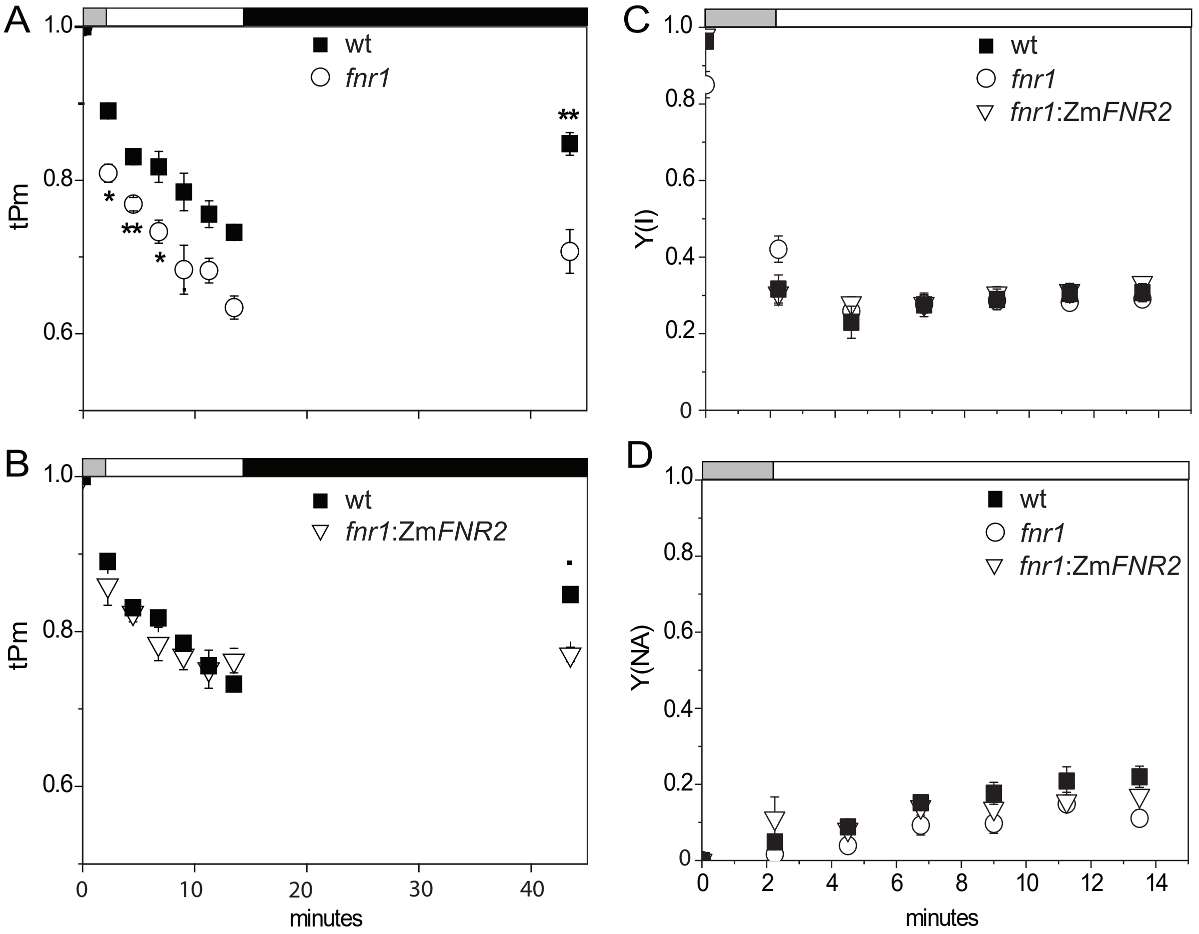

Supplemental Figure S2 Additional experiments on the impact of ferredoxin:NADP(H) oxidoreductase (FNR) tether associations on photosystem I (PSI) photoinactivation during high light exposure in Arabidopsis. Timing of changes in light intensity is indicated above the graph: the grey bar indicates a step-wise increase to first 543, then 692 µmol photon m^{-2} s^{-1} (1 minute each). The white bar indicates continuous illumination at 1385 µmol photon m^{-2} s^{-1}, and the black bar indicates a dark relaxation period. A Transient P700 maximum (tPm) in wt compared to *fnr1*. B, tPm in wt compared to *fnr1*:Zm*FNR2*. C, Effective quantum yield (Y(I)) of all genotypes. D, Acceptor limitation (Y(NA)) of all genotypes. Data shown are means ± S.E.M. (n≥3 individuals per genotype). Differences attributed by Student's T-test. P-value indicated by '***'< 0.001, '**'< 0.01, '*'< 0.05, '.'< 0.1.

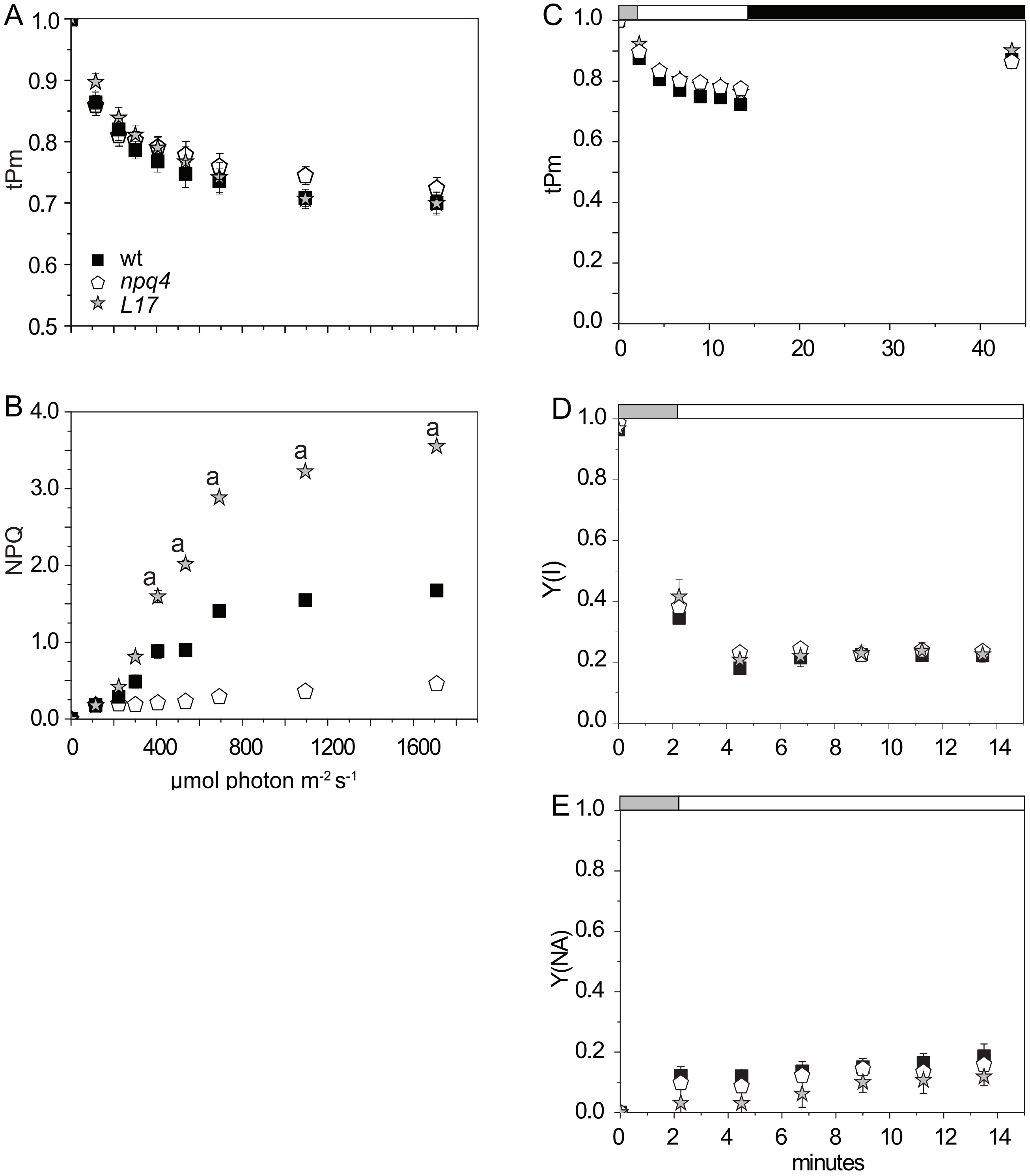

Supplemental Figure S3 Impact of altered non-photochemical quenching (NPQ) on photosystem I (PSI) inactivation over increasing light (A, B) and during sudden high light stress (C, D, E) in Arabidopsis. Genotypes are wt (black squares) the PsbS overexpressing genotype *L17* (grey stars), and the PsbS knock-out *npq4* (open pentagons). A, transient P700 maximum (tPm) over increasing light intensity. B, NPQ over increasing light intensity. C, tPm following sudden illumination at high light. Pm was determined after 30 minutes of dark adaptation. D, effective quantum yield of PSI (Y(I)). E, acceptor limitation at PSI (Y(NA)). In C, D and E timing of changes in light intensity is indicated above the graph: the grey bar indicates a step-wise increase to first 543, then 692 µmol photon m^{-2} s^{-1} (1 minute each). The white bar indicates continuous illumination at 1385 µmol photon m^{-2} s^{-1}, and the black bar indicates a dark relaxation period. Data are means ± S.E.M. (n≥3 individuals per genotype). No significant differences were found by Post Hoc honest significant difference Tukey Test and are indicated as the following letters: “a” for *L17* ≠ wt ≠ *npq4.*

.

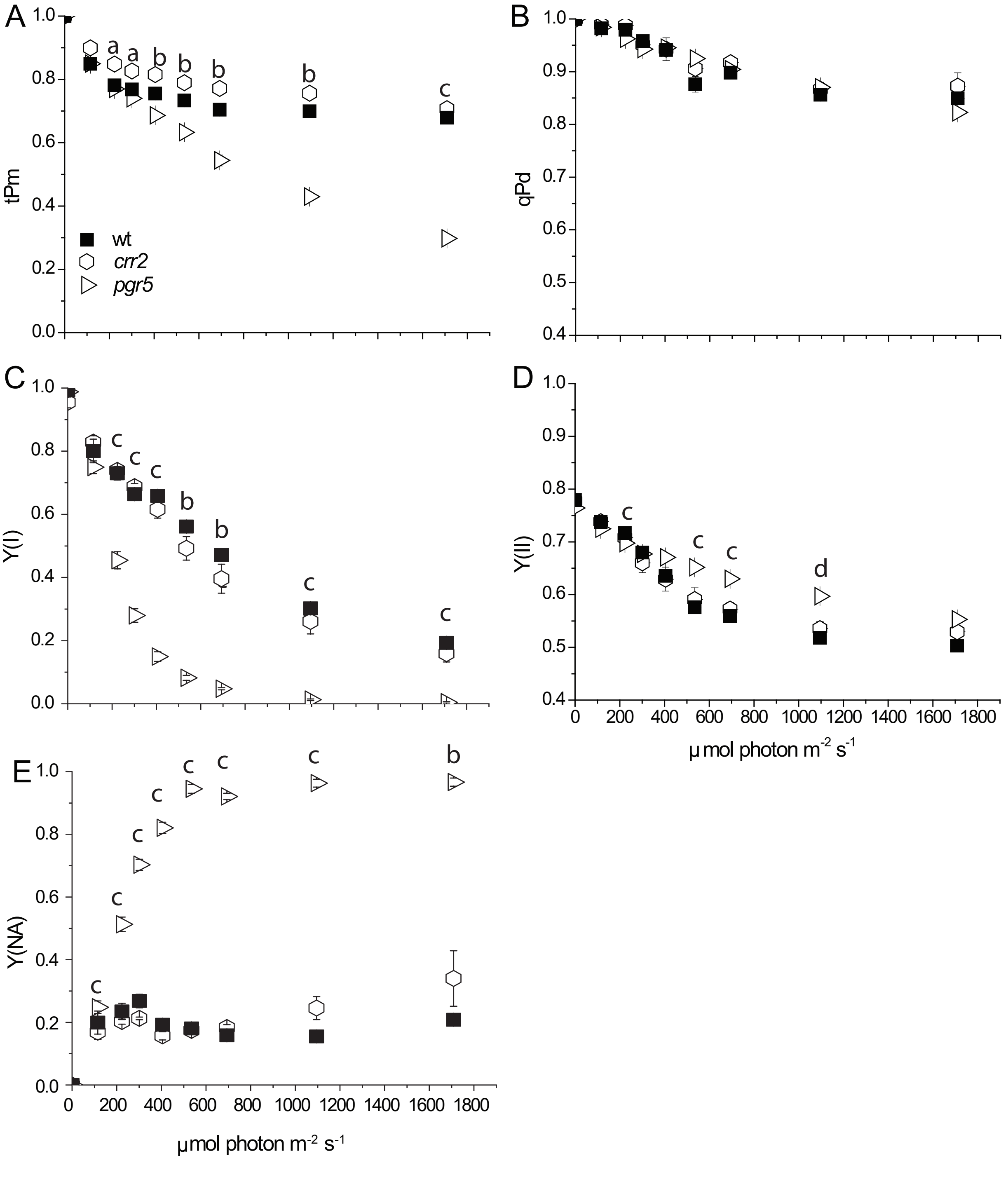

Supplemental Figure S4 Impact of two different cyclic electron flow (CEF) pathways on photoinactivation of photosystem I (PSI) and photodamage to photosystem II (PSII) during a gradual increase of light intensity. P700 absorption and chlorophyll a fluorescence measurements on Arabidopsis genotypes: wt (black squares), *pgr5* (open right pointing triangles) and *crr2* (open hexagons). A, transient maximum of Pm (tPm). B, effective quantum yield of PSI (Y(I)). C, acceptor limitation at PSI (Y(NA)). D, photochemical quenching in the dark (qPd). E, effective quantum yield of PSII (Y(II)). Data shown are means ± S.E.M. (n≥3 individuals per genotype). Significant differences were attributed by Post Hoc honest significant difference Tukey Test and are indicated by the following letters: “a” for wt and *pgr5* ≠ *crr2*; “b” for wt ≠ *pgr5* ≠ *crr2*; “c” for wt and *crr2* ≠ *pgr5*; “d” for wt ≠ *pgr5*.

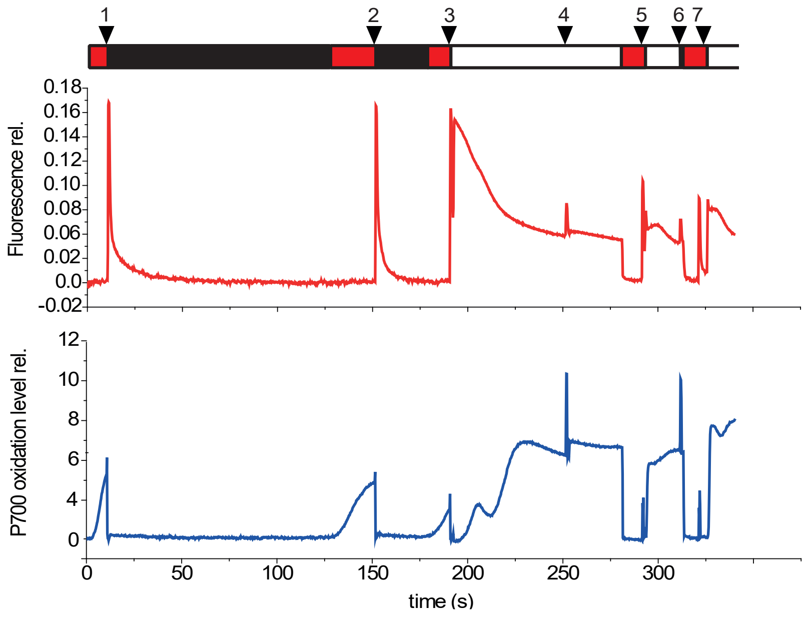

Supplemental Figure S5 Example traces of the initial section of a typical photosystem II (PSII) chlorophyll fluorescence (red trace) and photosystem I reaction center (P700) absorption (blue trace) simultaneous measurement with steps of increasing light intensity. The bar above the graph indicates changes in light and the timing of saturating pulses (downward arrows), far red illumination (red bars), darkness (black bars) and actinic light at 543, then 692 µmol photon m^{-2} s^{-1} (white bars). Pulses for parameter determination are indicated as follows: “1” initial P700 maximum (Pm) determination, “2” fluorescence maximum (Fm) determination, “3” and “7” were used for calculation of photochemical quenching in the dark (qPd), “4” is an additional Fm pulse, not used in this analysis, “5” was used for calculation of transient P700 maximum (tPm), “6” was used to calculation of non-photochemical quenching (NPQ), Effective quantum yield of PSI (Y(I)), acceptor limitation at PSI (Y(NA)).
